# Effects of Cable Sway, Electrode Surface Area, and Electrode Mass on Electroencephalography Signal Quality during Motion

**DOI:** 10.3390/s18041073

**Published:** 2018-04-03

**Authors:** Evangelia-Regkina Symeonidou, Andrew D. Nordin, W. David Hairston, Daniel P. Ferris

**Affiliations:** 1 J. Crayton Pruitt Family Department of Biomedical Engineering, University of Florida, Gainesville, FL 32611, USA; andrew.nordin@bme.ufl.edu (A.D.N.); dferris@bme.ufl.edu (D.P.F.); 2 Max Planck Institute for Biological Cybernetics, 72076 Tübingen, Germany; 3 International Max Planck Research School for Cognitive and Systems Neuroscience, 72074 Tübingen, Germany; 4 U. S. Army Research Laboratory, Aberdeen Proving Ground, Aberdeen, MD 21005, USA; william.d.hairston4.civ@mail.mil

**Keywords:** EEG, motion artifacts, electrode mass, electrode surface area, cable sway, signal to noise ratio, phantom head, locomotion

## Abstract

More neuroscience researchers are using scalp electroencephalography (EEG) to measure electrocortical dynamics during human locomotion and other types of movement. Motion artifacts corrupt the EEG and mask underlying neural signals of interest. The cause of motion artifacts in EEG is often attributed to electrode motion relative to the skin, but few studies have examined EEG signals under head motion. In the current study, we tested how motion artifacts are affected by the overall mass and surface area of commercially available electrodes, as well as how cable sway contributes to motion artifacts. To provide a ground-truth signal, we used a gelatin head phantom with embedded antennas broadcasting electrical signals, and recorded EEG with a commercially available electrode system. A robotic platform moved the phantom head through sinusoidal displacements at different frequencies (0–2 Hz). Results showed that a larger electrode surface area can have a small but significant effect on improving EEG signal quality during motion and that cable sway is a major contributor to motion artifacts. These results have implications in the development of future hardware for mobile brain imaging with EEG.

## 1. Introduction

Biopotential recordings are used to monitor physiological changes and have become an integral part of research and clinical diagnostics. To acquire these recordings, a variety of methods can be applied that can be narrowed down to two categories, non-invasive and invasive imaging. Electroencephalography (EEG), electrocardiography (ECG) and electromyography (EMG) are widely used recording techniques that can be non-invasive and capture neural, cardiac, and muscle activity, respectively. They fundamentally work the same way, by placing a metal electrode on the surface of the skin and creating an ionic connection between the skin and the electrode. This is transformed into electric current, reflecting the changes of potential taking place under the skin surface. EEG signals specifically capture changes of synchronized postsynaptic potentials of a large number of cortical pyramidal cells [1]. These changes can reflect neural excitation and different stages of phasic or tonic firing [2,3] and are important in clinical diagnostics such as epilepsy, in neuroscience research, as well as in technological breakthroughs such as brain computer interfaces (BCI) [4,5,6].

EEG electrodes can come in a variety of conductive metals and can be used in the presence or absence of an electrolyte—“wet” or “dry”. The most stable and widely used electrodes are Ag/AgCl electrodes, which are indirectly attached to the scalp with an electrolyte mediator. Ag/AgCl electrodes are preferred compared to others, due to their non-polarizable surface and resistive nature, which makes them capable of recording a wide range of frequencies (starting below 0.1 Hz) and less susceptible to motion artifacts [7,8].

Beyond the variety of sensors, various hardware and software have been developed over the years for data acquisition and processing both off-line and also in real time. This has led to advancements in the fields of rehabilitation, movement disorders, exercise physiology and BCI [9,10,11]. However, despite these advancements motion artifacts are still the biggest limiting factor in taking EEG research out of the lab and into the real world for more realistic, mobile behaviors [12]. 

Motion artifacts are especially problematic because of their large amplitude that masks the underlying neural signal of interest. Often seen as spiking or drifting in the EEG signal, they can have a variety of contributors [8,13]. One main contributor is electrode motion relative to the electrolyte and electrolyte motion relative to the skin. This causes changes in polarity at the electrode-electrolyte-skin-junction that appear as an artifact in the EEG [14]. To reduce this effect, electrodes can be mounted onto the head with a cap. Also, electrolyte gel should be applied correctly into the adhesive rings of the cap, or within the electrode well, and excessive amounts should be avoided. Alternatively, miniature electrodes can be used, since they have demonstrated higher robustness to accelerations and electrode-skin interface deformations during movements [15]. Cable sway in non-wireless systems also causes artifacts [16]. This can be attributed to the motion of the conductor within the magnetic field or to triboelectric noise that is caused by friction of the cable’s components [8,17]. To avoid that, special low noise components that reduce the friction within the cable can be used. When using passive electrodes, the electric signal gets amplified after it passes through the cable. Artifacts can be minimized, however, by using active electrodes, where the signal is pre-amplified at the electrode site [12,17,18]. Theoretically, signal quality can be generally improved with a larger electrode surface [17,19,20]. This is mostly attributed to an impedance decrease in the electrode-electrolyte-skin junction. Thus, by increasing the electrode surface without increasing the mass, motion artifacts might be reduced.

A previous study published from our lab compared the susceptibility of different commercially available hardware to motion artifacts [16]. Specifically, it was shown that the signal to noise ratio (SNR) decreased with increasing motion frequency. However, this decrease was the lowest for the wireless systems compared to the non-wireless one. Many studies have tried to identify components that contribute the most to motion artifacts [16,17,21,22,23,24]; however, to date none have done so by specifically modifying mass, surface area, or cable sway and quantifying their effect on the signal. In this paper, we extended on previous work and looked into three major contributors of motion artifacts:

(1) Electrode mass: We hypothesize that increasing the mass of the electrode can cause greater electrode displacement relative to the scalp during motion and, therefore, increase motion artifacts.
(2) Electrode surface area: We hypothesize that increasing the electrode surface area can lead to better signal quality during motion, because of the decrease in impedance of the electrode-electrolyte junction.
(3) Cable sway: We hypothesize that cable sway contributes the most to motion artifacts. Therefore, isolating cable motion from electrode motion will give us a better insight into motion artifact generation.

To test our hypotheses, we used a conductive gelatin phantom head embedded with antennas that generated a continuous sinusoidal electrical signal of a specific frequency on the scalp of the head. To evaluate the change in signal quality during motion, a hexapod motion platform was used to generate vertical sinusoidal displacements of 4 cm in different frequencies while we recorded EEG from the phantom. Signal to noise ratio was calculated from the signal recorded at each motion frequency and a ground truth baseline stationary condition. We chose a commercially available active EEG system that has been used the most in mobile human EEG studies.

## 2. Materials and Methods

### 2.1. The Phantom Head

**Figure 1:**
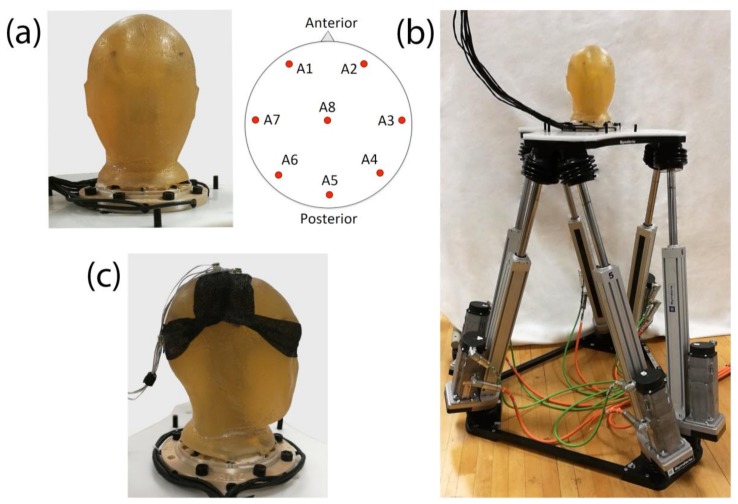
The Setup: (**a**) ballistics gel phantom head and antenna locations; (**b**) the motion hexapod platform with the head attached to it; (**c**) electrode placement on the ballistics gel phantom. Electrodes were placed on the top of the head (perpendicular to the motion) and on the back of the head (parallel to the motion). The CMS/DRL electrodes were also placed on the top of the head behind the recording electrodes.

For the experiments, we constructed a full sized human head model (Figure 1a), molded from ballistics gelatin (Vyse Gelatin Company, Schiller Park, IL, USA), as ballistics gelatin has been shown to be a reasonable surrogate for the conductive properties of human skin [25]. Similar to the previous study [16], the head contained a number of antennas embedded within its center with the capacity to generate electrical signals (Figure 1a). This way the electrical fields are transmitted through the gelatin medium out to the scalp, where electrodes were attached in a manner similar to typical human applications. The head mold and embedded base are from designs developed at the US Army Research Lab and are freely available upon request.

For the signal in the head, we used a single antenna and the TracerDAQ software (National Instruments, Austin, TX, USA) to generate a continuous sine wave at 7 Hz frequency. We were interested in examining the changes of the signal at the head surface. Therefore, for each experiment the antenna and the magnitude of the generated signal was chosen so that the signal recorded at each electrode was within the typical physiological amplitude range of human EEG.

### 2.2. The Motion Hexapod

To generate motion artifacts, we used a NOTUS motion hexapod (Symetrie, Nimes, France) (Figure 1b). Sinusoidal displacements with an amplitude of 4 cm were performed in five different frequencies: 1, 1.25, 1.5, 1.75 and 2 Hz. We chose this range of motion frequencies, because it covers the range of step frequencies used primarily during human walking. With the phantom head attached, we performed vertical displacements, since they have been shown to contribute the most to motion artifacts [16,26,27,28,29,30,31,32]. A 4 cm displacement was chosen, because it is a typical amount of vertical head motion that occurs during walking [27,30,33], which can also generate an evident amount of motion artifacts in the EEG signal [16]. A lower amplitude would not be adequate to produce observable motion artifacts on the signal and a higher amplitude would be outside the range of vertical head motions occurring during typical walking speeds. To examine cable sway, we detached the head from the platform and generated horizontal displacements with an amplitude of 4 cm to move the cables. We chose a 4 cm amplitude as it represents the minimum amount of cable sway that could be expected during mobile EEG data collections based on our past experiences [11,12,34,35,36,37,38,39,40]. The 4 cm cable sway also matched the displacement of the phantom head during the electrode mass and surface area experiments in order to allow for a better straightforward comparison. For all experiments, we recorded a baseline stationary condition with the hexapod’s motors turned on (“Stationary”) and the motors turned off (“Motors off”). The second was to quantify the electromagnetic-interference (EMI) of the hexapod’s motors on the EEG. For the motors off and the stationary conditions, we obtained recordings with and without the antenna generating signal. For the rest of the conditions, the antenna was not generating any signal.

### 2.3. EEG Acquisition

To record EEG, we used the BioSemi Active Two system and active, wet, flat type Ag/AgCl electrodes (BSM, BioSemi, Amsterdam, The Netherlands). We chose the BioSemi Active Two system because it is the most frequent EEG system used for mobile EEG studies on humans. We performed a Google Scholar search for EEG gait studies between 2010 and 2016, and the BioSemi Active Two was used in almost twice as many publications compared to other commercially available wired active electrode systems.

The BioSemi Active Two system uses a common mode sense (CMS) active electrode and a driven right leg (DRL) passive electrode. A feedback loop drives the voltage, where the electrodes are attached, close to the reference voltage of an analog to digital (AD) converter. This reduces the impedance of the DRL electrode compared with the ground electrodes used by other EEG systems [41]. In our experiments, we used either two or four recording electrodes to obtain the data used for analysis. One pair of electrodes was placed on the top of the head, right and left to the midline and perpendicular to the plane of motion. Another pair was placed on the back of the head, left and right to the midline and parallel to the plane of motion. We placed the CMS and DRL electrodes behind the first pair of electrodes, on a surface that was the closest to being perpendicular to the motion plane, which closely approximates the locations these electrodes are typically placed during human EEG recordings (Figure 1c). EEG was recorded at 512 Hz and a bandwidth between 0.01 and 256 Hz. The CMS electrode was used as the recording reference.

### 2.4. Electrode Mass

**Figure 2:**
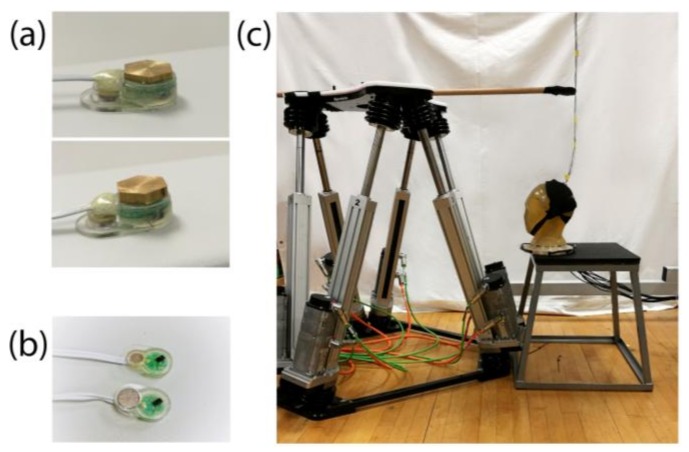
(**a**) Brass pieces used to double (top) and triple (bottom) the mass of the electrode for the electrode mass experiment; (**b**) the electrodes used for the surface area experiment. The standard unmodified electrode with the smaller surface area next to the modified electrode with the larger surface area; (**c**) setup of the cable sway experiment. The phantom was detached from the motion hexapod and was placed next to it. We secured a wooden rod on the motion hexapod and performed horizontal sinusoidal displacements to move the cables.

We wanted to examine if increasing the electrode mass would decrease the SNR during motion. For increasing the electrode mass, we used brass since it has high-density and is non-ferromagnetic. We custom cut brass pieces and attached them with double sided tape to the recording electrodes to double or triple their mass. The recording electrode was 670 mg when detached from its cable and the brass pieces had an average mass of 671 ± 28 mg for the doubled mass condition and 1380 ± 18 mg for the tripled mass condition (Figure 2a).

The recording protocol consisted of 4 blocks. Within each block, every mass condition (1 × Electrode Mass, 2 × Electrode Mass and 3 × Electrode Mass) was recorded for each motion condition (“Motors off”, “Stationary”, 1, 1.5, 1.25, 1.5 and 1.75 Hz). There were 4 trials of each combined mass-motion condition. Within each mass condition, the motion conditions were randomized. Each brass piece was randomly attached to an electrode and the pieces were interchanged within each block. The trial duration was 60 s. Three blocks were used for the analysis. We excluded one block, because not all electrode signals of interest were recorded for all motion conditions.

We secured the electrodes on the phantom head (Figure 1c) with the Andover Powerflex Sports Tape (Ithaca Sports, Ithaca, NY, USA) and used double-sided electrode stickers (Cortech Solutions, Inc., Wilmington, NC, USA) to attach the electrodes to the tape. Electrolyte gel (SignaGel, Cortech Solutions, Inc.) was applied in the holes that were cut out of the tape to allow for the electrode to come in contact with the head surface.

### 2.5. Electrode Surface Area

We wanted to see if the SNR decrease would be smaller for a larger electrode surface. To test that, we modified the BioSemi flat type recording electrodes. The original 4 mm diameter Ag/AgCl pellet was removed and replaced with a 6 mm diameter Ag/AgCl pellet (Figure 2b).

The recording protocol consisted of four blocks. Within each block, we recorded all motion conditions once (“Motors off”, “Stationary”, 1, 1.5, 1.25, 1.5 and 1.75 Hz), resulting in a total of four trials per condition. We randomized the motion conditions within each block and each trial was 60 s long.

We used Powerflex Sports Tape to secure the electrodes on the head (Figure 1c) and double-sided stickers to attach the electrodes on the tape. Electrolyte was applied within the holes of the tape, which were adjusted to the electrode’s surface area. Two electrodes with small surface area (4 mm diameter) and two electrodes with large surface area (6 mm diameter) were used. We placed a large and a small surface electrode on the top and on the back of the head, perpendicular and parallel to the motion. 

### 2.6. Cable Sway

To examine the contribution of cable sway on motion artifacts, we detached the head from the motion hexapod and performed horizontal instead of vertical displacements to move the cables. We wanted to reproduce the motion that the cables experienced during the electrode mass and electrode surface recordings, without any accelerations acting on the electrodes. To move the cables, we used a wooden rod secured to the motion hexapod (Figure 2c) and performed horizontal sinusoidal displacements with a 4 cm amplitude. The rod was attached to the cables to ensure sway towards one direction, as well as a constant displacement of 4 cm across all motion frequencies. The cable length was 155 cm. Similar to the previous experiments we tested seven different conditions (“Motors off”, “Stationary”, 1, 1.5, 1.25, 1.5 and 1.75 Hz).

We used the ballistics gel head and attached the electrodes as described in the electrode mass and surface area experiment. We used additional Powerflex Sports Tape to secure the electrodes and cables on the head for electrode-cable strain relief. Four blocks were performed, resulting in a total of 4 trials for each motion condition. A trial was 60 s long and motion conditions were randomized within each block. We excluded from the analysis the two electrodes placed on the top of the head, since the recorded amplitude of the antenna’s generated signal was below an acceptable level. Additionally, since the head wasn’t moving, we didn’t expect differences in forces acting on the electrodes depending on their head placement. We kept the electrodes that were placed on the back of the head for the analysis.

### 2.7. EEG Analysis

We processed EEG using MATLAB 2016a (MathWorks, Inc., Natick, MA, USA) and the EEGLAB toolbox [42]. From each trial of 60 s, 30 s were used for the analysis. The first 15 s and last 15 s of the recorded data were discarded. Subsequently, the data was bandpass filtered between 0.5 and 59 Hz with the fir-filter function. To calculate the SNR, we divided the log root mean square (RMS) of the stationary condition when the antenna was generating signal by every condition when the antenna was not generating any signal (1):

(1) $$\mathrm{SNR}=\;20\log10\;(\frac{\;\mathrm{RMS}\;\mathrm{signal}\;\mathrm{on}\;\mathrm{stationary}}{\mathrm{RMS}\;\mathrm{signal}\;\mathrm{off}\;\mathrm{condition}})$$

To evaluate the EMI from the motion hexapod’s motors, we also calculated the SNR by dividing the signal on and off conditions with the hexapod’s motors off. We only used one antenna to generate signal during each experiment and we placed the electrodes on two different locations (top and back of the head). Therefore, there were differences in the SNR across electrodes that arose from the electrodes’ proximity to the antenna. To minimize this effect, we normalized the SNR for each trial of each condition to the average SNR of the “Motors off” condition. This way we had a uniform baseline value across electrodes, focusing on the SNR changes due to motion. We also averaged the normalized SNR values of the neighboring electrodes for each head location (top of the head and back of the head) for the mass and cable sway recordings since we didn’t expect differences in head side. The normalized SNR values were averaged within each condition for plotting.

### 2.8. Statistical Analysis

We analyzed the resulting normalized SNR values with the SPSS software (SPSS Inc., Chicago, IL, USA). A repeated measures ANOVA was performed for each experiment. For the mass recordings the factors were (a) mass (1 × Electrode Mass, 2 × Electrode Mass and 3 × Electrode Mass), (b) motion frequency (“Motors off”, “Stationary”, 1, 1.25, 1.5, 1.75 and 2 Hz) and (c) head location (top of the head, back of the head). For the electrode surface area recordings, the factors were (a) electrode surface area (small surface, large surface), (b) motion frequency (“Motors off”, “Stationary”, 1, 1.25, 1.5, 1.75 and 2 Hz) and (c) head location (top of the head, back of the head). Last, for the cable sway experiment, the factor was motion frequency (“Motors off”, “Stationary”, 1, 1.25, 1.5, 1.75 and 2 Hz). We applied Greenhouse-Geisser correction whenever the assumption of sphericity was violated, and report *p*-values based on the corrected degrees of freedom.

## 3. Results

### 3.1. Electrode Mass

**Figure 3:**
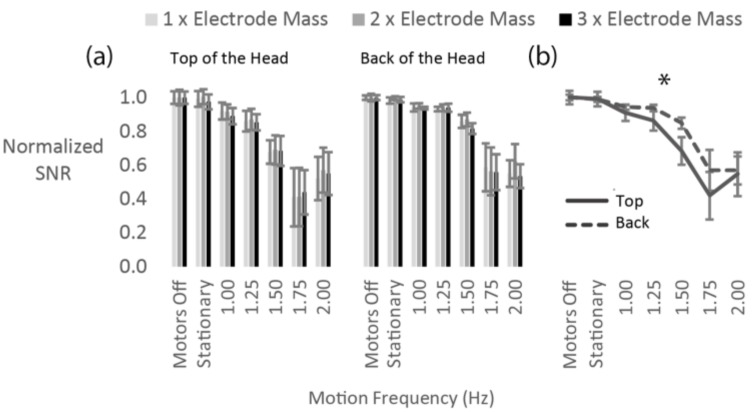
(**a**) Normalized signal to noise ratio (SNR) values during motion for each mass condition for electrodes on the top and the back of the head; (**b**) normalized SNR values averaged across all mass conditions for all frequencies for the top and the back of the head. There was no general effect of electrode mass on the SNR. There was a significant main effect of motion frequency and an interaction between head location and motion frequency. The plotted error bars represent the standard error of the mean.

Contrary to our hypothesis, increasing the mass of the electrode by three-fold did not affect the SNR across all electrodes (Figure 3). Statistically, there was no significant effect of electrode mass on the normalized SNR (Figure 3a). However, we found a significant effect of motion frequency (F_(1.0, 2.0)_ = 23.9, *p* = 0.038) and a significant interaction between motion frequency and head location (F_(1.5, 2.9)_ = 30.2, *p* = 0.012) as seen in Figure 3b.

### 3.2. Electrode Surface Area

**Figure 4:**
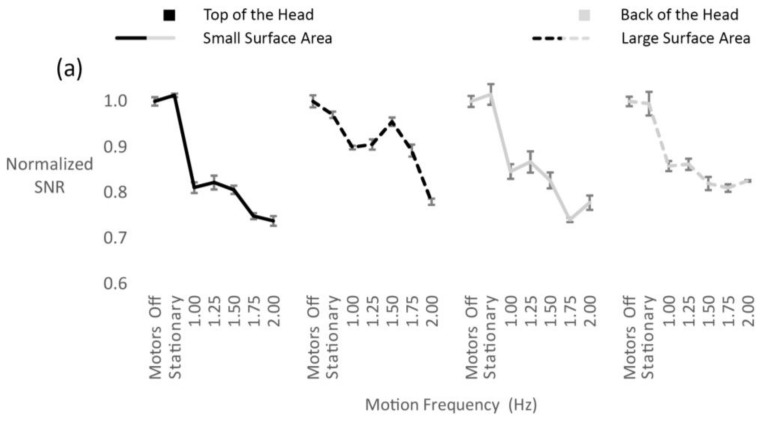

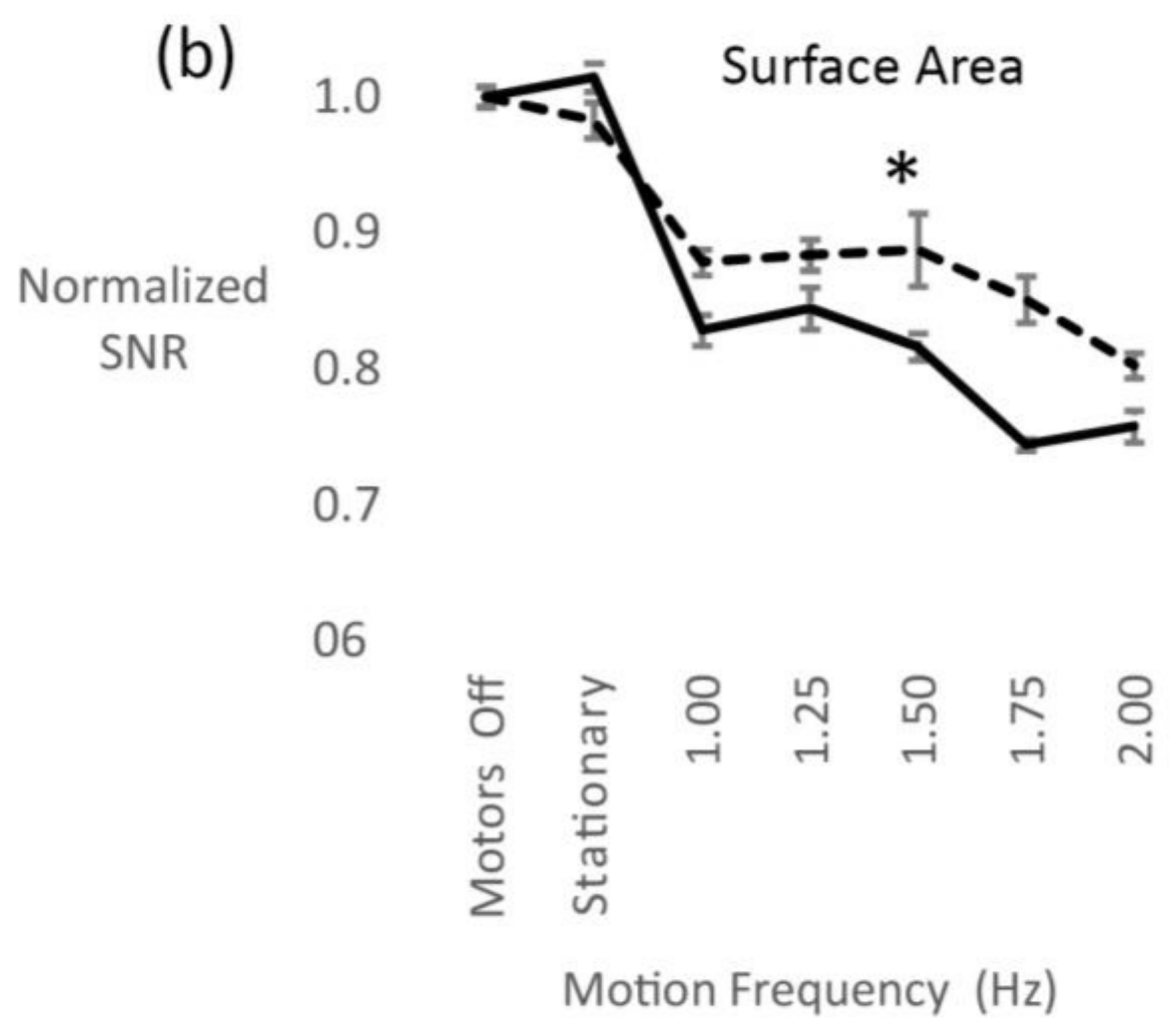
(**a**) Normalized signal to noise ratio (SNR) values for each electrode surface area and head location relative to motion; (**b**) average normalized SNR of the large surface area electrodes (dotted lines) and small surface area electrodes (solid lines). A larger electrode surface area resulted in a slightly better SNR. Error bars represent the standard error of the mean.

Increasing the surface area of the electrodes had a positive effect on the SNR (Figure 4). We observed a significant main effect of electrode surface area (F_(1, 3)_ = 54.5, *p* = 0.005) and motion frequency (F_(2, 6.1)_ = 112.3, *p* < 0.001) (Figure 4a). Additionally, there was a significant interaction between surface area and motion frequency (F_(2.3, 6.9)_ = 52.8, *p* < 0.001) (Figure 4b). Significant interactions were also found between head location and motion frequency (F_(1.7, 5.2)_ = 15.1, *p* = 0.007), as well as head location and surface area (F_(1, 3)_ = 36.3, *p* = 0.009) . Specifically, electrodes on the top of the head were affected less by motion artifacts than electrodes on the back of the head, especially the electrode with the large surface area (Figure 4a). The large surface area electrode on the top of the head exhibited a higher SNR when compared with the large surface area electrode on the back of the head across most frequencies. This was shown by a significant three-way interaction between surface area, head location and motion frequency (F_(1.7, 5.1)_ = 26.2, *p* = 0.002) (Figure 4a).

### 3.3. Cable Sway

**Figure 5:**
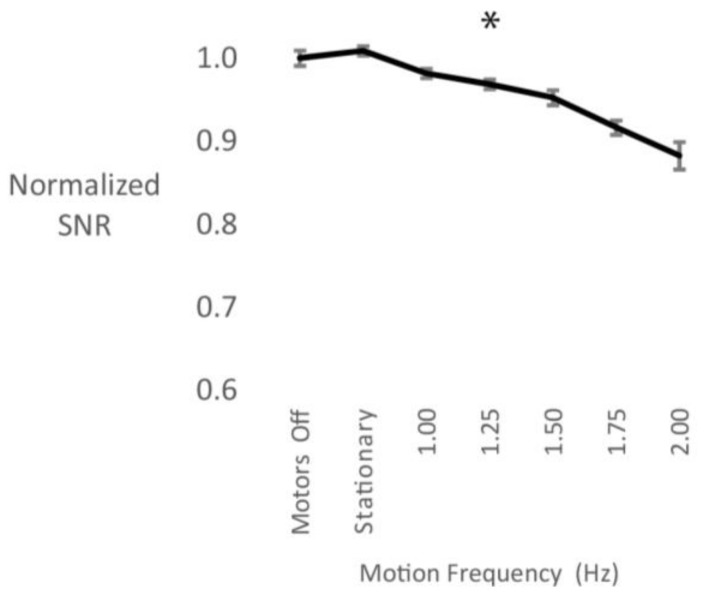
Normalized signal to noise ratio (SNR) values during cable sway. SNR decreased with increasing motion frequency. Error bars represent the standard error of the mean.

Cable sway had a large effect on the motion artifact. There was a main effect of motion frequency (F_(2.2, 6.6)_ = 24.8, *p* = 0.001), where higher cable motion frequencies decreased the normalized SNR (Figure 5).

## 4. Discussion

We found that increasing cable sway led to a decrease in SNR, and that a larger electrode surface area had a small but significant effect on improving signal quality during motion. Electrode mass did not affect the SNR for the motion frequencies we tested. This experiment is the first to quantify the effect of three hardware components, namely (a) mass, (b) electrode surface area and (c) cable sway on motion artifacts on a commercially available EEG system. Using the novel ballistics gel head phantom allowed us to compare the recorded EEG signal with a ground truth signal that was broadcast within the head. By placing the phantom head on a motion platform, we could accurately control the motion of the head and EEG system for the experiments. We added in cable motion to our study because our past experience collecting data on walking and running humans suggested that cable motion was an important factor in the signal quality. These findings are the first to demonstrate that cable sway is a major contributor to the generation of motion artifacts. The results also indicate that surface area in an active electrode EEG system can have a significant effect on the signal quality.

### 4.1. Electrode Mass

Micro-movements of EEG electrodes relative to the scalp surface can result in increased artifacts [14,16]. Ödman and Oberg [43] suggested that movement induced potentials are mostly attributed to polarity changes between electrode and skin. More massive electrodes would require greater forces to maintain their position relative to the scalp surface than less massive electrodes. As a result, it was possible that the electrodes with greater mass would have larger displacement relative to the scalp surface. This should have led to polarity changes in the electrode-electrolyte-skin junction, which were expected to be the strongest for the electrodes on the back of the head. We did not see any changes in SNR when we doubled or tripled the mass of the electrodes. Further increasing the mass might have had a quantifiable effect on the SNR, but currently available electrode systems are becoming smaller and lighter. The three-fold range of electrode masses tested seems to be the most realistic for current commercially available systems. Further experimentation could be done by significantly decreasing the mass of the currently commercially available electrodes to see if the SNR was better at reduced electrode masses. For the mass experiment, we observed a reduction in SNR with increasing motion frequency as expected from our previous work [16]. We also saw an interaction between head location and motion frequency, with the electrodes on the top of the head showing larger SNR decrease than the electrodes on the back of the head. This effect was mostly pronounced in the 1.5 Hz condition and is in agreement with previous observations [31]. 

### 4.2. Electrode Surface Area

A larger electrode surface area resulted in a higher SNR, with an improvement between 5% and 10% for the intermediate motion frequencies. It has already been shown that electrocardiogram (ECG) electrodes with a small recording surface area increase the electrode-skin impedance and are more susceptible to noise and motion artifacts [20,44,45]. Lee and colleagues developed flexible ECG electrodes with a larger recording surface that had the capability to bend around the body, thus increasing the area of electrode-skin-contact. These electrodes led to an increase in SNR compared to rigid electrodes with smaller recording surfaces [45]. Additionally, Simakov and Webster [17] showed that electrode conductance increases with surface area, leading to reduced motion artifacts. Our results were able to support those findings. The difference in SNR we observed, between the modified and unmodified electrodes, however, was small relative to the change in surface area, which was doubled. Additionally, the electrode with the increased surface area on the back of the head did not show any benefit compared to the unmodified electrode at the same head location. This might be attributed to the fact that modification led to a change in depth of the electrode gel cavity, which in turn may have resulted in decreased stability of contact between electrode and scalp, which was more pronounced on the back of the head. This might have masked the beneficial effect of the increased electrode surface area on the SNR at this head location. 

### 4.3. Cable Sway

Cable sway resulted in a 10% overall decrease in the SNR. Also, this decrease followed a linear pattern compared to the SNR decrease seen in the surface area experiment that was most pronounced between the stationary and the 1.0 Hz motion frequency condition (Figure 4). For wired systems, there is evidence that cable sway is the major contributor to motion artifacts in mobile EEG [8,12,17]. It has been suggested, however, that this effect has been minimized through active shielding of the cables and pre-amplification of the signal at the electrode side with the use of active electrodes [12,17,34,46]. In this study we used an active electrode system, and our results suggest that pre-amplification of the signal still results in significant signal quality decrease with cable motion.

Wireless systems eliminate the problem of cable sway artifacts, but data showing their superiority for mobile EEG measurements are lacking. In stationary EEG experiments, both wired and wireless systems yield similar quality results [47,48,49]. Experiments involving locomotion have been inconclusive. Debener and colleagues were able to reliably obtain a P300 component during an outdoor walking experiment with a low-cost, wireless EEG system [50]. However, for their BCI application, classification accuracy was significantly higher when participants were seated compared to when they were moving. In a previous study of our lab, we compared signal quality of different commercially available systems during motion [16]. We found that the wireless EEG system resulted in the lowest SNR decrease with increasing motion frequency and displacement compared to the wired systems. This was, however, because the wireless system had a very low performance during baseline, which didn’t deviate significantly with increasing motion. There need to be additional studies on wireless systems examining signal quality during head motion in order to determine if wireless systems offer advantages over wired systems in terms of signal quality. Therefore, this study focused on a commercially available wired system. It is probable that, in the future, wireless EEG systems will be able to substitute wired systems for experiments involving locomotion. However, our observations have shown that there are still considerations concerning signal quality, user comfort, and electrode density that need to be addressed [16,48,51].

A number of growing applications rely on clean EEG signal, both in clinical and non-clinical environments. BCI supported gait rehabilitation and exoskeletons were developed to aid individuals with mobility impairments [52,53]. Also, EEG recordings are used to study performance in moving athletes [54]. These are highly dynamic applications and are extremely susceptible to cable motion. Currently, our results suggest that for the EEG system that has been most often used in studies on mobile EEG (i.e., BioSemi Active Two), the cable sway issue is likely the factor that contributes the most to motion artifacts during human walking. To be able to collect reliable signals in dynamic settings, future EEG hardware development needs to examine how to reduce cable sway for wired systems. 

## 5. Conclusions

This study provided evidence that both electrode surface area and cable sway can influence motion artifacts during mobile EEG recordings. We found that electrode surface area can have a small but significant effect on improving EEG signal during motion, and that cable sway largely contributes to motion artifacts. These results provide better insight into the causes of motion artifacts, which can help improve future development of better and more robust hardware.
